# Erector Spinae Plane Block in Post-thoracotomy Pain Management: A Case Series

**DOI:** 10.7759/cureus.65360

**Published:** 2024-07-25

**Authors:** Afnan Amjad, Faraz Mansoor

**Affiliations:** 1 Anesthesia and Critical Care, Shaukat Khanum Memorial Cancer Hospital and Research Centre, Peshawar, PAK

**Keywords:** ultrasound-guided catheter placement, regional anesthesia, esophagectomy, post-thoracotomy pain management, erector spinae plane block

## Abstract

Post-thoracotomy pain management remains challenging, with thoracic epidurals traditionally considered the gold standard. Erector spinae plane blocks have emerged as a potential alternative, offering simplicity and a favorable safety profile. This case series presents three patients undergoing a hybrid Ivor Lewis esophagectomy who received erector spinae plane blocks for postoperative analgesia. Ultrasound-guided erector spinae plane catheters were inserted pre-anesthesia, with infusions managed postoperatively based on individual pain scores. All patients achieved effective pain control, enabling early mobilization. This suggests erector spinae plane blocks as a viable option for post-thoracotomy pain management.

## Introduction

Pain management strategies in the field of anesthesiology have undergone a paradigm shift, with the increasing popularity of plane blocks as an alternative to more invasive methods such as thoracic epidurals [1]. This case series investigates the evolving landscape of pain management, focusing on the application of erector spinae plane blocks in post-thoracotomy patients. The primary advantages of plane blocks include their ease of insertion, minimal impact on vital signs, and reduced risk of complications, making them an appealing choice over traditional epidurals [2].

Research has highlighted the efficacy of plane blocks, particularly in the context of chronic post-thoracotomy pain [3]. Effective pain management is integral to early patient mobilization and discharge, with the added benefit of diminished opioid consumption and a subsequent reduction in opioid-related side effects, notably respiratory depression and hemodynamic instability [4].

While thoracic epidurals have long been considered the gold standard for post-thoracotomy pain control, the emergence of plane blocks, specifically erector spinae blocks, has presented a compelling alternative. This newer technique, described by Forero et al. in 2016 [5], involves the administration of local anesthetics through the erector spinae plane, targeting the dorsal and ventral rami via the costotransverse foramen. Continuous infusion through a catheter in this plane facilitates sustained pain relief.

In our clinical setting, traditionally favoring thoracic epidurals, we present three cases where erector spinae plane blocks were employed for post-thoracotomy pain control. This choice was informed by the ease of insertion and served as a secondary approach following failed epidural attempts, aligning with the growing evidence of the promising outcomes associated with erector spinae blocks.

## Case presentation

This case series presents three patients with American Society of Anesthesiologists (ASA) physical status II, diagnosed with gastro-esophageal junction carcinoma. All patients were scheduled for hybrid Ivor Lewis surgery (two-stage esophagectomy). Written informed consent was obtained from each patient, who also received detailed education regarding the erector spinae plane block. The procedure was performed in the operating theater immediately prior to anesthesia induction. Standard monitoring, including pulse oximetry, electrocardiography, and noninvasive blood pressure measurement, was established before the procedure. Pre-anesthesia, erector spinae plane catheter insertion was performed uniformly with patients in a seated position, using a B. Braun epidural kit (Melsungen, Germany) with an 18G Tuohy needle under linear probe ultrasound guidance. The procedure was performed at the T5 vertebral level, with the needle directed in a cephalocaudal direction. An in-line approach was employed to contact a transverse process with the Tuohy needle, after which the plane was opened using 5-7 mL of normal saline. The epidural catheter was inserted to a depth of 12 cm in all cases. As all patients were known cases of gastro-esophageal junction carcinoma, they had almost the same body mass index (BMI).

Following ultrasound confirmation of catheter placement, the patients were positioned supine for anesthesia induction. Induction was achieved with propofol (titrated to effect), muscle relaxation with atracurium (weight-based dosing), and anesthesia maintenance with isoflurane. Fentanyl (100 μg) was administered intravenously at induction, and morphine (5 mg) was given immediately before incision. A radial arterial line was inserted in all patients for continuous blood pressure monitoring. The hybrid Ivor Lewis procedure was conducted in two stages: abdominal esophageal resection followed by thoracic resection. Erector spinae plane infusion (bupivacaine 0.125% at 5 mL/hour) was initiated immediately before thoracotomy. At the end of the surgery, local infiltration with 20 mL of 0.25% bupivacaine was performed at all surgical sites. Postoperative pain was assessed using a numeric rating scale (0-10) upon emergence. All patients received a standardized analgesic regimen consisting of paracetamol 1000 mg thrice daily and tramadol 50 mg thrice daily.

Case 1

A 46-year-old female with controlled asthma as her sole comorbidity underwent surgery 12 weeks after laparoscopic staging for cancer. The procedure lasted seven hours. Erector spinae plane catheter infusion was initiated intraoperatively at 5 mL/hour. Upon emergence, the patient reported no pain. Eight hours postoperatively, the pain score increased to 4/10, necessitating an increase in the infusion rate to 7 mL/hour. At 15 hours post-surgery, the patient was successfully mobilized without pain and remained pain-free subsequently.

Case 2

A 54-year-old female with no comorbidities underwent surgery eight weeks after staging laparoscopy. The procedure duration was seven hours and 20 minutes. Erector spinae plane infusion commenced at 5 mL/hour intraoperatively. Upon emergence, the patient reported mild pain (4/10). The erector spinae plane infusion rate was increased to 8 mL/hour, resulting in effective pain relief. The patient was mobilized 10 hours postoperatively and remained pain-free.

Case 3

A 54-year-old female with hepatitis C (normal liver function tests) underwent surgery 10 weeks following staging laparoscopy. The procedure lasted 10 hours. Erector spinae plane infusion was initiated at 5 mL/hour intraoperatively. The patient reported no pain upon emergence. Mobilization occurred at eight hours postoperatively, at which point the patient experienced mild pain (4/10). The erector spinae plane infusion rate was increased to 7 mL/hour, providing effective analgesia thereafter.

## Discussion

Pain management following thoracotomy poses significant challenges, traditionally addressed by thoracic epidurals but increasingly supplemented or replaced by regional techniques such as erector spinae plane blocks [6]. Our case series contributes to the evolving understanding of erector spinae plane blocks in post-thoracotomy pain control, highlighting their efficacy and potential advantages over epidural anesthesia.

The primary benefit of erector spinae plane blocks lies in their simplicity and safety profile [7], as evidenced by our experiences with three patients undergoing hybrid Ivory Lewis surgeries. This technique, first described by Forero et al. [5], involves injecting a local anesthetic into the fascial plane adjacent to the erector spinae muscles, providing effective analgesia by blocking both the dorsal and ventral rami of spinal nerves. Our approach involved standardized insertion procedures using ultrasound guidance, ensuring accurate placement and subsequent infusion through an indwelling catheter.

In our cases, erector spinae plane blocks facilitated immediate postoperative pain relief, allowing early patient mobilization and reducing the need for systemic opioids. This aligns with existing literature emphasizing the role of regional anesthesia in improving postoperative outcomes by minimizing opioid-related adverse effects such as respiratory depression and delayed recovery [8]. Notably, all patients in our series reported satisfactory pain control postoperatively, with adjustments in infusion rates effectively managing breakthrough pain episodes.

While our institution traditionally favored thoracic epidurals for thoracotomy patients, the successful implementation of erector spinae plane blocks in these cases underscores their viability as an alternative or adjunctive method. The choice to employ erector spinae plane blocks, particularly following failed epidural attempts, reflects a growing trend in anesthesiology toward optimizing patient care through tailored, minimally invasive techniques [9].

Future studies could further explore the long-term benefits of erector spinae plane blocks in terms of chronic pain management and patient satisfaction beyond the immediate postoperative period. Comparative studies against traditional methods such as thoracic epidurals would also provide valuable insights into the optimal approach for post-thoracotomy pain management in different patient populations.

## Conclusions

In conclusion, erector spinae plane block, in these cases, illustrates its promising role in enhancing perioperative care for thoracotomy patients. As anesthesia practices continue to evolve, erector spinae plane blocks represent a valuable addition to the armamentarium of pain management strategies, offering effective analgesia with favorable safety and recovery profiles.
